# Cognitive, behavioral, and affective mechanisms underlying the efficacy of Applied Relaxation in reducing psychopathological symptoms: A randomized controlled trial

**DOI:** 10.1016/j.xjmad.2024.100055

**Published:** 2024-02-22

**Authors:** Eva Asselmann, Frank Rückert, Hanna Kische, Monique Zenker, Lars Pieper, Katja Beesdo-Baum

**Affiliations:** a Department of Psychology, Institute for Mental Health and Behavioral Medicine, HMU Health and Medical University, Potsdam, Germany; b Behavioral Epidemiology, Institute of Clinical Psychology and Psychotherapy, Faculty of Psychology, TUD, Dresden University of Technology, Dresden, Germany

**Keywords:** Depression, Anxiety, Stress, Health promotion, Mediator, Stress reduction

## Abstract

**Background:**

This study examined the cognitive, behavioral, and affective mechanisms underlying the efficacy of Applied Relaxation (AR) in reducing psychopathological symptoms. AR is a cognitive-behavioral technique that allows for rapid relaxation at the first sign of stress or tension in daily life.

**Methods:**

A randomized controlled trial was conducted in 277 adults (18–55 years) with elevated symptoms of depression, anxiety, or stress but without a 12-month DSM-5 mental disorder at study entry. Participants were randomized to an intervention group receiving AR training (10 weeks, N = 139) and an assessment-only control group (N = 138). Mental health outcomes (depressive, anxiety, and stress symptoms) and potential cognitive (self-efficacy, perceived control), behavioral (coping behaviors) and affective (positive and negative affect) mediators of the intervention efficacy were assessed at baseline and post-assessment in both groups.

**Results:**

Structural equation models indicated that baseline to post reductions in psychopathological symptoms due to AR partially passed through less avoidance-oriented and less other dysfunctional coping (proportion of total effect mediated; ratio of indirect to total effect: resignation: 55.0%, rumination: 27.9%, escape: 27.4%, aggression: 21.3%), less negative affect (46.3%), more positive affect (41.8%), lower external control beliefs (14.5%), and higher self-efficacy (13.3%).

**Conclusions:**

Our results suggest that improvements in cognitive, behavioral, and affective mediator variables partially explain the intervention efficacy of AR in improving mental health.

**Trial registration and data statement:**

The study protocol has been pre-registered on ClinicalTrials.gov (NCT03311529). The study protocol, minimum dataset, and analysis codes are available at OpARA - Open Access Repository and Archive. Supplementary materials (e.g., the course manual and additional training materials) are available on request from the last author (katja.beesdo-baum@tu-dresden.de).

## Highlights

• We tested potential mediators of the efficacy of Applied Relaxation training (AR).
• A randomized controlled trial with an intervention and a control group was conducted.
• AR led to reductions in psychopathological symptoms.
• AR led to improvements in potential cognitive, behavioral, and affective mediators.
• These improvements partially explained the positive mental health effects of AR.

## 1 Introduction

Mental disorders are highly prevalent [62], [69], cause significant impairment [68], and result in enormous direct and indirect societal costs [28]. Therefore, a major challenge in clinical psychology is to develop targeted early interventions to prevent the development of full-threshold mental disorders at their earliest stages.

One particularly promising preventive intervention is Applied Relaxation (AR; [12]. Originally developed for the treatment of anxiety disorders, AR allows for rapid relaxation in 20–30 s at the first sign of tension or stress in everyday life [50]. Based on Progressive Muscle Relaxation, AR training teaches people to relax gradually in shorter intervals (and without prior tension), to recognize the first signs of tension/stress in everyday life, and to relax not only in stress-free but also in stressful situations to prevent a vicious cycle of symptoms that escalate over time.

### 1.1 Efficacy of AR

Previous research has shown that AR can effectively reduce psychopathological symptoms in patients with various mental disorders and somatic diseases. For example, AR could reduce anxiety symptoms in patients with generalized anxiety disorder [5], [26], [27], [33], [53], panic disorder [51], [56], [57], agoraphobia [37], [54], [57], social phobia [21], [48], [49], and specific phobias [20], [55]. Furthermore, AR reduced pain symptoms in patients with chronic or recurrent headache [2], [63], [65], migraine [44], [47], neck and back pain [1], [29], long-standing pain [38], [43], [60], [64], and tinnitus [4], [17]. The technique also led to improvements in relaxation [19], general anxiety [36], and nonspecific physical symptoms and body pain [35] in students, reductions in state/trait anxiety and perceived stress in pregnant women [10], and reductions in anxiety in athletes [46]. Taken together, these findings illustrate the potential of AR as a transdiagnostic approach for the treatment and prevention of various mental disorders, (psycho)somatic complaints, and related health problems.

Building on these previous findings, we conducted a randomized controlled trial in adults with elevated depressive, anxiety, or stress symptoms but without any 12-month mental disorder at study entry to examine the broader mental health effects of AR. Results showed that all psychopathological symptoms, assessed with conventional questionnaires and ecological momentary assessments in daily life, decreased more in the intervention vs. control group from baseline to post-assessment [8], [12]. In addition, AR resulted in higher self-efficacy, higher internal control beliefs, more approach-oriented (i.e., relaxation and positive self-instruction) and less avoidance-oriented (i.e., escape and avoidance) coping, and more positive affect, assessed with ecological momentary assessments in daily life [8].

### 1.2 Potential mechanisms of action

Cognitive-behavioral models suggest that psychophysiological tension plays an important role in the development of mental disorders and is part of a vicious cycle of symptoms that escalate over time [11], [42]. Relaxation interventions aim to break such a vicious cycle by reducing psychophysiological tension and inducing relaxation [16]. Psychophysiological relaxation, in turn, can lead to positive cognitive, behavioral, and affective changes and reductions in psychopathological symptoms [8].

As suggested by previous research [31], such cognitive, behavioral, and affective changes might play a particularly important role in the context of AR. AR is conceptualized as a behavioral coping technique that allows individuals to manage rather than avoid stressful situations [50]. Because individuals learn to approach and manage rather than avoid stressful situations, AR training might lead to less avoidance-oriented coping (e.g., escape, avoidance, and distraction) and less other dysfunctional coping (e.g., resignation, rumination, aggression, or medication) but more approach-oriented coping (e.g., relaxation, positive self-instruction, situation control, and reaction control; [8]. At the cognitive level, the ability and experience to successfully cope with stress might lead to higher self-efficacy, higher internal control beliefs, and lower external control beliefs [8], [9]. At the emotional level, these positive experiences and stress reductions in daily life might lead to more positive and less negative affect [8].

Previous research based on patient samples partially supports the idea that specific cognitive, behavioral, and psychophysiological mechanisms might underlie the efficacy of AR: Among patients with generalized anxiety disorder, reductions in worry due to AR partially accounted for subsequent reductions in somatic symptoms and vice versa [23], [24], suggesting that improvements in cognitive and somatic outcomes might reinforce each other over time. In addition, improvements in decentering preceded improvements in anxiety symptoms [30], and improvements in experiential avoidance preceded improvements in worry and quality of life [26]. However, among patients with long-standing pain, there was no evidence that psychological inflexibility, catastrophizing, or pain intensity mediated the effects of AR on pain symptoms over the course of the intervention [38]. Among adults with elevated psychopathological symptoms, improvements in salivary cortisol secretion did not mediate improvements in psychopathological symptoms due to AR [39].

In summary, these preliminary findings suggest that specific cognitive and behavioral changes might underlie the efficacy of AR. However, most previous research has focused on specific patient samples and mechanisms that are particularly relevant to these groups (e.g., pain in patients with long-standing pain or worry in patients with generalized anxiety disorder). Therefore, additional research is needed to examine whether changes in broader cognitive, behavioral, and affective processes targeted by AR (e.g., self-efficacy and perceived control, approach- and avoidance-oriented coping, and positive/negative affect) underlie the efficacy of AR in reducing psychopathological symptoms in individuals with elevated psychopathological symptoms. Individuals with elevated psychopathological symptoms are at increased risk of progressing to full-threshold psychopathology [6], [7], [13] and thus are particularly likely to benefit from indicated preventive interventions and AR training [12], [22].

### 1.3 Aims and hypotheses

Using data from a randomized controlled trial in individuals with elevated psychopathological symptoms but without a 12-month mental disorder at study entry, this study investigated the cognitive, behavioral, and affective mechanisms underlying the efficacy of AR training. Individuals with 12-month mental disorders were not included, as the trial aimed to test the efficacy of AR as an indicated preventive intervention in prospectively preventing (rather than treating) mental disorders [12]. Specifically, we examined whether improvements in psychopathological symptoms due to AR (from baseline to post) passed through changes in self-efficacy and perceived control, approach- and avoidance-oriented coping, and positive and negative affect.

The following hypotheses were tested: Main effects: From baseline to post, individuals in the intervention vs. control group experience greater reductions in psychopathological symptoms as well as greater improvements in potential cognitive mediators (higher self-efficacy, higher internal control beliefs, lower external control beliefs), behavioral mediators (less avoidance-oriented and other unfavorable coping behaviors; more approach-oriented coping), and affective mediators (more positive and less negative affect). Mediation effects: Intervention-induced changes in psychopathological symptoms partially pass through intervention-induced changes in these cognitive, behavioral, and affective variables (indirect effects).

Indicators of avoidance-oriented coping include escape, avoidance, and distraction. Indicators of other unfavorable coping behaviors include resignation, rumination, aggression, and medication. Indicators of approach-oriented coping include relaxation, positive self-instruction, situation control, and reaction control.

## 2 Materials and methods

### 2.1 Design

A parallel group randomized controlled superiority trial with an intervention group receiving group-based AR training and an assessment-only control group was conducted from June 2016 to November 2019 at the Institute of Clinical Psychology and Psychotherapy, TUD—Dresden University of Technology, Germany [12]. Participants were assigned to the intervention or control group by balanced randomization [1:1 ratio] based on computer-generated permutated blocks.

The study included a baseline assessment, a post-assessment, and a 12-month follow-up assessment. The post-assessment was conducted immediately after the intervention in the intervention group and after a similar period of time in the control group (i.e., approximately 3 months after baseline).

The study protocol was pre-registered at ClinicalTrials.gov (NCT03311529). The study protocol, minimum data set, and analysis codes will be made available at OpARA - Open Access Repository and Archive after formal acceptance. Supplementary materials (e.g., the course manual and additional training materials) are available on request from the last author (katja.beesdo-baum@tu-dresden.de).

The study was approved by the Ethics Committee of the TUD—Dresden University of Technology (EK508112015). All participants gave written informed consent after being fully informed about the study and were paid € 8.50 per hour for their participation in the post- and follow-up assessments.

### 2.2 Sample characteristics and attrition

Detailed information on the study flow chart, including sociodemographic and clinical characteristics of the sample and reasons for dropout at different stages of the study, has been presented previously [12]. Individuals who did not participate in the post- and 12-month follow-up assessments and individuals who continued to participate in the study did not differ from each other on most demographic variables, dimensional psychopathological symptoms and any subthreshold mental disorder at baseline, and group status. However, noncompleters of dimensional symptom scales reported fewer children (*p* = .011) and worse financial situation (*p* = .048) than did completers [12].

### 2.3 Participants

Participants were recruited from the general population and cooperating institutions in the Dresden area through flyers, advertisements, and media articles. Individuals interested in the study were screened for initial inclusion criteria (see below) via a secure website of the TUD—Dresden University of Technology. Individuals who met these criteria were invited to participate in an entry exam that included a standardized diagnostic interview modified for DSM-5 (DIA-X-5; [34] to assess lifetime and 12-month mental disorders. Further details of the interview procedures in this study have been presented previously [12].

Inclusion criteria were at least mild depressive, anxiety, or stress symptoms (depression score ≥5, anxiety score ≥4, or tension/stress score ≥8 on the Depressive Anxiety Stress Scale, DASS-21; [45] and age 18–55 years. The main exclusion criterion was a 12-month diagnosis of a mental disorder, including somatic symptom and related disorders (i.e., somatic symptom disorder and illness anxiety disorder), anxiety disorders (i.e., panic disorder, generalized anxiety disorder, social anxiety disorder, agoraphobia, and separation anxiety disorder), depressive disorders (i.e., major depression and persistent depressive disorder/dysthymia), bipolar disorders (i.e., bipolar I disorder and bipolar II disorder), posttraumatic stress disorder, and substance use disorders (i.e., alcohol use disorder, medication use disorder, and illicit substance use disorder) as assessed by the DIA-X-5. Other exclusion criteria were psychotic symptoms and acute suicidality. It was planned to withdraw subjects who developed psychosis or acute suicidality during the study and refer them to treatment. However, no such events occurred. Participants were required not to receive any pharmacological or psychological interventions at study entry, but were free to receive such (additional) interventions during the study.

### 2.4 Intervention

The AR intervention was manualized according to the procedures introduced by Öst [50], [52] and delivered in a group format with approximately 10 participants per course. Each course consisted of 10 instructor-led sessions (each lasting approximately one hour) with the following content: (1) Psychoeducation (i.e., information about the rationale, goals, and procedures of AR) and progressive muscle relaxation, (2) release-only relaxation I (i.e., direct muscle relaxation without prior tension), (3) release-only relaxation II (i.e., direct muscle relaxation without prior tension, repetition of the previous class session), (4) cue-controlled relaxation (i.e., relaxation with a personal cue word), (5) differential relaxation I (i.e., relaxation with eyes open and with movement of the eyes, head, arms or legs, as well as in sitting position), (6) differential relaxation II (i.e., relaxation while standing and walking), (7) rapid relaxation (i.e., shortened cue-controlled relaxation [20–30 s] in daily life), (8) AR - imagery practice (i.e., rapid relaxation at the first sign of tension triggered by an imaginary scenario), (9) AR – real-life practice (i.e., transfer to real-life situations typically associated with psychophysical, emotional, cognitive, or behavioral symptoms of tension or distress), (10) AR in real life, relapse prevention, and closure.

The course sessions were accompanied by weekly homework (i.e., daily practice of relaxation exercises with corresponding notes in a relaxation practice diary). Moreover, participants were instructed to document daily stress episodes and associated psychophysical, cognitive, behavioral, and emotional symptoms in a tension/distress diary (to train early recognition of early signs of tension and distress). Homework assignments were prepared and discussed in each course session. All course instructors (*N* = 4) had a background in psychology (i.e., undergraduate psychology or a bachelor’s or master’s degree in psychology) and were trained in AR by the principal investigators (KBB and EA) and supervised throughout the study. The AR course manual and materials are available on request from KBB or EA.

### 2.5 Measures and potential mediators

At baseline and post-assessment, self-report measures of psychopathological symptoms and potential mediators of the intervention efficacy were assessed with widely used and established instruments using tablet computers. Each scale was answered on a sliding scale from 0 to 100.

*Psychopathological symptoms.* Depressive, anxiety, and stress symptoms in the past 2 weeks were assessed using the 21-item DASS-21 [45], labeled from 0 = “never” to 100 = “very often / most of the time” (7 items per subscale). Example items are “I couldn’t seem to experience any positive feelings at all” for depressive symptoms, “I felt scared without any good reason” for anxiety symptoms, and “I found it hard to wind down” for stress symptoms.

The reliability and validity of the DASS-21 have been shown to be high in clinical and non-clinical samples [3], [18], [32], [58], [59]. In this sample, Cronbach's alphas were α = .86 at baseline and α = .89 at post for depressive symptoms, α = .79 at baseline and α = .82 at post for anxiety symptoms, and α = .88 at baseline and α = .89 at post for stress symptoms.

*Potential cognitive mediators.* Self-efficacy was measured using the 3-item General Self-Efficacy Short Scale (“I can rely on my own abilities in difficult situations”, “I am able to solve most problems on my own”, and “I can usually solve even challenging and complex tasks well”, labeled from 0 = “does not apply to me at all” to 100 = “applies to me completely”). The scale has been shown to have good psychometric properties [14]. In this sample, Cronbach's alphas were α = .91 at baseline and α = .94 at post.

Internal and external control beliefs were assessed using a 4-item scale [40], of which 2 items measure internal control beliefs (“I'm my own boss”, “If I work hard, I will succeed”), and 2 items measure external control beliefs (“Whether at work or in my private life: What I do is mainly determined by others”, “Fate often gets in the way of my plans”), labeled from 0 = “does not apply to me at all” to 100 = “applies to me completely”. The psychometric properties of this scale have been shown to be good [40]. In this sample, Cronbach's alphas were α = .71 at baseline and α = .83 at post for internal control beliefs and α = .56 at baseline and α = .79 at post for external control beliefs.

*Potential behavioral mediators.* Avoidance-oriented coping (escape, avoidance, and distraction), other unfavorable coping behaviors (resignation, rumination, aggression, and medication), and approach-oriented coping (relaxation, positive self-instruction, situation control, and reaction control) were assessed with 66 items from the Stress Processing Questionnaire (“Stressverarbeitungsfragebogen”; [25]. In this questionnaire, participants are asked to indicate how likely they are (from 0 = “not at all” to 100 = “very likely”) to react in certain ways when something or someone upsets or upsets them or throws them off balance (6 items per subscale). Example items are “I tend to flee” for escape, “I avoid such situations from now on” for avoidance, “I do something to distract myself” for distraction, “I tend to give up quickly” for resignation, “I keep thinking about it” for rumination, “I get angry” for aggression, “I drink a glass of beer, wine, or liquor first” for medication, “I try to relax my muscles” for relaxation, “I tell myself I will get through it” for positive self-instruction, “I take steps to eliminate the cause” for situation control, and “I tell myself to pull myself together” for reaction control.

Reliability and validity information has been reported previously [25]. In the current sample, Cronbach's alphas were α = .91 at baseline and α = .94 at post for escape, α = .90 at baseline and α = .88 at post for avoidance, α = .75 at baseline and α = .85 at post for distraction, α = .89 at baseline and α = .90 at post for resignation, α = .96 at baseline and α = .97 at post for rumination, α = .90 at baseline and α = .90 at post for aggression, α = .68 at baseline and α = .65 at post for medication, α = .87 at baseline and α = .95 at post for relaxation, α = .89 at baseline and α = .93 at post for positive self-instruction, α = .78 at baseline and α = .80 at post for situation control, and α = .79 at baseline and α = .81 at post for reaction control.

*Potential affective mediators.* Positive and negative affect in the past week was assessed using the 20-item Positive and Negative Affect Schedule [67]. This scale consists of 10 single-adjective items for positive affect (e.g., “active”, “proud”, “enthusiastic”) and 10 single-adjective items for negative affect (e.g., “ashamed”, “nervous”, “jittery”), labeled from 0 = “not at all” to 100 = “extremely”. The reliability and validity of this scale have been shown to be good [41]. Cronbach's alphas were α = .91 at baseline and α = .93 at post for positive affect and α = .85 at baseline and α = .86 at post for negative affect.

### 2.6 Statistical analyses

Information on sample size calculation focusing on the primary outcome analyses has been published previously [12].

First, changes in psychopathological symptoms (DASS-21 total score) and potential mediators from baseline to post were compared in the intervention vs. control group. To do so, associations between group (dummy variable, coded as 1 in the intervention group and 0 in the control group) and the difference score (post minus baseline) of each outcome were tested using structural equation modeling in Stata 15 [61]. Second, total group effects and indirect group effects (i.e., the effect on the outcome passing through the respective potential mediator) on symptom changes from baseline to post were calculated.

The analyses were adjusted for sex, age at baseline, and baseline scores of the respective outcome and/or mediator variable at baseline [66]. Full information maximum likelihood estimation was used to handle missing data (e.g., at post vs. baseline). The alpha level was set at .05. The p-values of the mediator effects were controlled for false discovery rates using the Benjamini-Hochberg method [15]. We controlled for all mediators at once (i.e., 16 effects in Table 2 and 12 effects in Table 3).

**Table 1:** Means and standard deviations for individual outcomes at baseline and post in the intervention (N = 139) and control (N = 138) group (total: N = 277).

	Baseline (N = 277)				Post (N = 229)			
	Intervention group (N = 139)		Control group (N = 138)		Intervention group (N = 110)		Control group (N = 119)	
Outcome	M	SD	M	SD	M	SD	M	SD
Psychopathological symptoms	26.51	10.67	27.45	10.92	20.12	9.33	27.10	11.75
Self-efficacy	74.18	15.17	72.72	16.25	79.54	14.56	74.39	15.27
Internal control beliefs	74.72	13.96	71.41	13.68	77.17	14.63	72.59	14.91
External control beliefs	29.72	15.42	30.62	14.93	24.35	17.86	29.78	19.53
Avoidance-oriented coping								
Escape	36.63	19.80	36.23	19.25	24.97	19.93	37.19	18.77
Avoidance	45.99	17.85	45.37	18.47	40.44	17.39	44.06	16.28
Distraction	49.98	14.07	49.96	12.54	46.03	17.54	52.49	12.98
Other dysfunctional coping								
Resignation	32.01	16.99	35.26	17.82	22.86	16.91	36.18	17.04
Rumination	64.35	20.38	65.27	22.12	54.34	22.05	65.13	22.37
Aggression	35.52	20.33	37.98	18.02	28.04	18.55	38.27	18.08
Medication	7.98	8.70	8.91	9.96	5.47	6.39	8.25	9.14
Approach-oriented coping								
Relaxation	40.79	16.82	40.29	18.88	67.51	17.15	38.63	18.66
Positive self-instruction	60.86	16.94	60.67	15.80	62.98	22.04	61.15	16.10
Situation control	63.79	13.47	62.64	14.07	62.01	14.68	63.77	12.00
Reaction control	60.69	15.39	58.33	13.90	60.09	17.20	58.96	13.09
Positive affect	48.93	13.55	49.46	14.10	58.46	15.05	50.61	13.88
Negative affect	22.95	12.18	23.91	11.45	18.41	10.49	23.92	13.11

*Notes.* M, mean; SD, standard deviation.

**Table 2:** Group effects (intervention vs. control group) on changes in psychopathological symptoms and potential mediators from baseline to post (N = 277).

	Group				
Outcome	β	95% CI		p	p_cor_
Psychopathological symptoms	-0.62	-0.83	-0.41	<.001	–
Potential mediators	β	95% CI		p	p_cor_

Self-efficacy	0.24	0.06	0.43	.011	.016
Internal control beliefs	0.21	-0.02	0.44	.075	.092
External control beliefs	-0.38	-0.64	-0.12	.004	.006
Avoidance-oriented coping					
Escape	-0.62	-0.81	-0.43	<.001	<.001
Avoidance	-0.22	-0.42	-0.02	.029	.039
Distraction	-0.50	-0.76	-0.24	<.001	<.001
Other dysfunctional coping					
Resignation	-0.62	-0.80	-0.43	<.001	<.001
Rumination	-0.52	-0.72	-0.32	<.001	<.001
Aggression	-0.40	-0.56	-0.24	<.001	<.001
Medication	-0.23	-0.38	-0.08	.004	.006
Approach-oriented coping					
Relaxation	1.55	1.33	1.76	<.001	<.001
Positive self-instruction	0.09	-0.15	0.34	.457	.487
Situation control	-0.17	-0.37	0.04	.117	.134
Reaction control	-0.03	-0.26	0.20	.800	.800
Positive affect	0.56	0.33	0.79	<.001	<.001
Negative affect	-0.41	-0.63	-0.18	<.001	<.001

*Notes.* β = beta coefficient from structural equation modeling. CI, confidence interval; p, p-value.

**Table 3:** Total group effects and indirect group effects (i.e., through the potential mediator) on changes in psychopathological symptoms from baseline to post (N = 277).

	Changes in psychopathological symptoms from baseline to post										
	Indirect group effect					Total group effect					
Mediator variable	b	95% CI		p	p_cor_	b	95% CI		p	p_cor_	%
Self-efficacy	-0.08	-0.16	-0.01	.023	.035	-0.60	-0.81	-0.39	<.001	<.001	13.33
External control beliefs	-0.09	-0.16	-0.02	.014	.022	-0.62	-0.83	-0.41	<.001	<.001	14.52
Avoidance-oriented coping											
Escape	-0.17	-0.27	-0.07	.001	.002	-0.62	-0.83	-0.41	<.001	<.001	27.42
Avoidance	-0.02	-0.05	0.02	.277	.300	-0.62	-0.83	-0.41	<.001	<.001	n.s.
Distraction	-0.02	-0.07	0.03	.444	.444	-0.61	-0.82	-0.40	<.001	<.001	n.s.
Other dysfunctional coping											
Resignation	-0.33	-0.46	-0.20	<.001	<.001	-0.61	-0.82	-0.40	<.001	<.001	55.00
Rumination	-0.17	-0.26	-0.08	.001	.002	-0.61	-0.82	-0.40	<.001	<.001	27.87
Aggression	-0.13	-0.21	0.04	.003	.006	-0.61	-0.82	-0.40	<.001	<.001	21.31
Medication	-0.05	-0.10	-0.00	.071	.092	-0.61	-0.82	-0.40	<.001	<.001	n.s.
Approach-oriented coping											
Relaxation	-0.18	-0.38	0.02	.077	.092	-0.61	-0.82	-0.40	<.001	<.001	n.s.
Positive affect	-0.23	-0.35	-0.11	<.001	<.001	-0.55	-0.79	-0.31	<.001	<.001	41.82
Negative affect	-0.25	-0.39	-0.10	.001	.002	-0.54	-0.77	-0.30	<.001	<.001	46.30

*Notes.* β = beta coefficient from structural equation modeling. CI , confidence interval; p, p-value.

All baseline and difference scores were standardized based on the pooled standard deviation in the intervention and control group at baseline.

## 3 Results

As previously presented [12], 277 individuals (intervention group: *N* = 139; control group: *N* = 138) participated at baseline and 229 (intervention group: *N* = 110; control group: *N* = 119) participated at post. For psychopathological symptoms, there were 277 observations at baseline and 227 observations at post. For each mediator variable, there were 277 observations at baseline and 228 observations at post.

The sample consisted of 190 women and 87 men aged 18–55 years at baseline (*M* = 34.41, *SD* = 10.66). As previously reported [12], sociodemographic characteristics at baseline (i.e., sex, age, education, employment, social class, financial situation, marital status, living arrangements, number of children, and nationality) did not differ between groups. In addition, there were no significant group differences in psychopathological symptom levels and rates of subthreshold mental disorders at baseline.

In the intervention group, 105 of 139 participants (75.5%) completed the AR training with *M* = 7.82 sessions attended (ranging from 4 to 10 sessions). Only 18 (13.0%) did not attend any course sessions, and 16 (11.5%) terminated the training early after attending *M* = 3.25 sessions (ranging from 1 to 6 sessions).

### 3.1 Main effects

Means and standard deviations for individual outcomes at baseline and post in the intervention and control group are shown in Table 1. As shown in Table 2, participants in the intervention vs. control group experienced not only greater reductions in psychopathological symptoms from baseline to post (*β* = −0.62). They also experienced greater improvements in potential mediators, including more approach-oriented coping (relaxation: *β* = 1.55), less avoidance-oriented coping (escape: *β* = −0.62, distraction: *β* = −0.50, avoidance: *β* = −0.22), less other dysfunctional coping (resignation: *β* = −0.62, rumination: *β* = −0.52, aggression: *β* = −0.40, medication: *β* = −0.23), more positive affect (*β* = 0.56), less negative affect (*β* = −0.41), lower external control beliefs (*β* = −0.38), and higher self-efficacy (*β* = 0.24).

### 3.2 Mediation effects

**Fig. 1:**
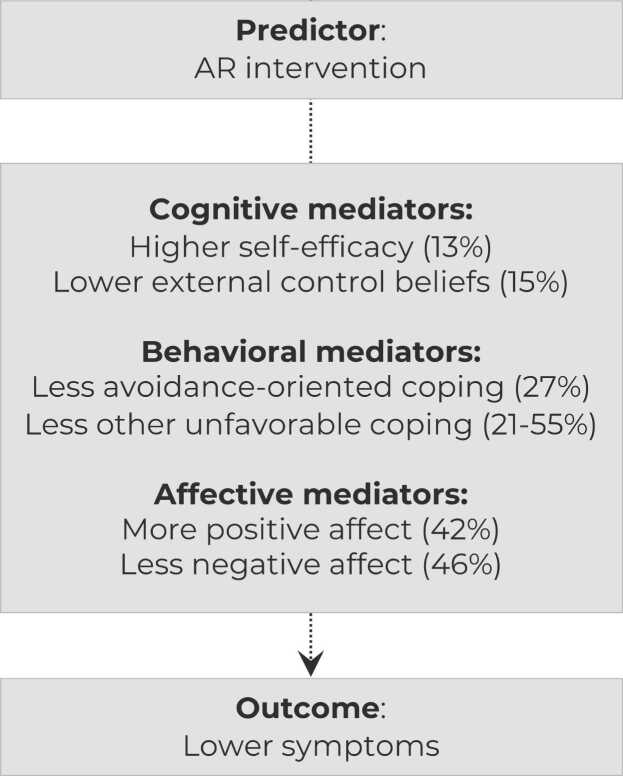
Mediation (i.e., indirect) effects.

As shown in Table 3 and Fig. 1, the calculation of total and indirect effects revealed that the group effects (intervention vs. control group) on changes in psychopathological symptoms from baseline to post partially passed through less avoidance-oriented and less other dysfunctional coping (proportion of total effect mediated; ratio of indirect to total effect: resignation: 55.0%, rumination: 27.9%, escape: 27.4%, and aggression: 21.3%), less negative (46.3%) and more positive (41.8%) affect, lower external control beliefs (14.5%), and higher self-efficacy (13.3%). These results suggest that these variables mediated the efficacy of the AR intervention.

## 4 Discussion

The aim of this study was to examine potential mechanisms underlying the efficacy of AR training. Consistent with our hypotheses and previous evidence [12], we found that individuals in the intervention vs. control group experienced greater reductions in psychopathological symptoms and greater improvements in potential cognitive, behavioral, and affective mediators from baseline to post.

Cognitive improvements due to AR included decreases in external control beliefs and increases in self-efficacy. Behavioral improvements due to AR included increases in approach-oriented coping (relaxation) and decreases in avoidance-oriented coping (escape, distraction, and avoidance) and other dysfunctional coping (resignation, rumination, aggression, and medication). Affective improvements included more positive and less negative affect.

Participants in the intervention group might have learned to manage rather than avoid stressful situations, which could explain the behavioral effects. These new coping strategies might have been more effective in reducing stress, which could explain the affective effects. In addition, the experience of successfully coping with stress might have increased self-efficacy and reduced external control beliefs. These findings support the idea of AR as a behavioral coping technique that allows individuals to manage rather than avoid stressful situations [50].

Estimation of mediation effects indicated that substantial portions of the observed symptom improvements passed through less avoidance-oriented and less other dysfunctional coping, less negative and more positive affect, and, to a lesser extent, lower external control beliefs and higher self-efficacy. These findings are consistent with our hypotheses and suggest that the positive mental health effects of AR were at least partially due to affective, behavioral, and cognitive changes. These findings are partially consistent with previous findings on conceptually related constructs. For example, there was evidence that reductions in worry mediated reductions in somatic symptoms [23], [24] or that improvements in decentering and experimental avoidance preceded improvements in anxiety and worry [26], [30] due to AR. However, this study was the first to examine the mediating role of different stress coping strategies, self-efficacy and perceived control, and affective well-being.

One possible explanation for the fact that the behavioral and affective mediation effects were stronger than the cognitive mediation effects could be that individuals trained in AR tend to intuitively relax in stressful situations and thus feel better immediately. However, AR users might be less likely to consciously reflect on these behavioral and emotional processes, which could explain why smaller indirect cognitive effects were found.

### 4.1 Strengths and limitations

This study has several strengths: The efficacy of AR in reducing psychopathological symptoms in individuals with elevated anxiety, depressive, or stress symptoms was assessed in a randomized controlled trial. Psychopathological symptoms and potential mediators of the intervention efficacy were assessed before and after the intervention using well-established scales.

However, our study is not without limitations: First, diagnostic information was assessed by self-report, which might differ from clinical expert ratings and behavioral observations (e.g., regarding actual coping behaviors in daily life).

Second, there was no active control group. Therefore, we cannot exclude the possibility that the intervention effects on psychopathological symptoms and potential mediators were due to nonspecific factors such as treatment expectations and personal contact with study staff and other participants during the group intervention, which might have led to increased social support. However, as previously reported [8], individuals in the intervention group (vs. control group) were more likely to relax in daily life after the intervention, indicating actual behavioral changes due to AR training.

Third, mediator and outcome variables were assessed simultaneously, so it was not possible to rigorously test whether intervention-induced changes in the mediator variables occurred temporally prior to intervention-induced changes in the outcome variables.

Fourth, the study was conducted in a convenient sample of young and middle-aged adults (18–55 years, 68.6% females) from the Dresden area, Germany. Therefore, generalizability to children, adolescents, and older adults and to other regional groups might be limited.

## 5 Conclusions

We found that AR training not only reduced psychopathological symptoms, but also led to positive cognitive, behavioral, and affective changes that partially explained the positive mental health effects. These findings underscore that AR is a behavioral coping technique that allows individuals to manage (rather than avoid) stressful situations [50]. As stress coping plays a central role in (almost) every mental disorder, our study highlights the utility of AR as a transdiagnostic approach in the treatment and prevention of various mental disorders and (psycho)somatic symptoms.

In our study, the mental health effects of AR were partially mediated by changes in stress coping, self-efficacy, and perceived control. Thus, one could speculate whether AR interventions are particularly effective when they explicitly inform about favorable and unfavorable stress coping strategies and when they strengthen self-efficacy and internal locus of control (e.g., through additional cognitive-behavioral techniques). Further studies are needed to test whether such additional intervention elements enhance the efficacy of AR training.

Moreover, further research would be useful to examine in more detail the temporal associations between changes in potential mediators and changes in psychopathological symptoms: How do intervention-induced cognitive, behavioral, and affective changes translate into better mental health, and how do these spillovers unfold over time? To address these questions, studies with high-frequency ecological momentary assessments during the intervention would be particularly useful.

## Ethical standards

The authors declare that all procedures contributing to this work comply with the ethical standards of the relevant national and institutional committees on human experimentation and with the Helsinki Declaration of 1975 as revised in 2013. The study was approved by the ethics committee of the TUD – Dresden University of Technology (EK508112015).

## Funding source

This randomized controlled trial (“Effectiveness and underlying mechanisms of Applied Relaxation as indicated preventive intervention in subjects at increased risk for mental disorders”) was funded by the German Research Foundation (Deutsche Forschungsgemeinschaft, DFG), project no. AS 497/1-1. The study protocol and data are available at OpARA—Open Access Repository and Archive (https://opara.zih.tu-dresden.de/xmlui/?locale-attribute=en). The funding source had no role in the design, conduct, analysis or reporting of the study.

## Role of the funding source

All authors had complete freedom to direct the analysis and its reporting without any influence from sponsors. There was no editorial direction or censorship by any sponsor.

## CRediT authorship contribution statement

**Eva Asselmann:** Conceptualization, data curation, formal analysis, funding acquisition, investigation, methodology, project administration, resources, supervision, validation, visualization, writing - original draft, writing - review & editing. **Frank Rückert:** Data curation, software, validation, writing - review & editing; **Hanna Kische:** Writing - review & editing. **Monique Zenker:** Data curation, investigation, project administration. **Lars Pieper:** Methodology, resources, software. **Katja Beesdo-Baum:** Conceptualization, data curation, funding acquisition, methodology, project administration, supervision, validation, writing - review & editing.

## Declaration of Competing Interest

The authors declare no potential conflicts of interest with respect to the research, authorship, and/or publication of this article
